# Associations between socio-economic factors and alcohol consumption: A population survey of adults in England

**DOI:** 10.1371/journal.pone.0209442

**Published:** 2019-02-04

**Authors:** Emma Beard, Jamie Brown, Robert West, Eileen Kaner, Petra Meier, Susan Michie

**Affiliations:** 1 Research Department of Behavioural Science and Health, University College London, London, England; 2 Institute of Health & Society, Newcastle University, Newcastle upon Tyne, England; 3 ScHARR, University of Sheffield, Sheffield, England; 4 Research Department of Clinical, Educational and Health Psychology, University College London, London, England; Universidad Miguel Hernandez de Elche, SPAIN

## Abstract

**Aim:**

To gain a better understanding of the complex relationships of different measures of social position, educational level and income with alcohol consumption in England.

**Method:**

Between March 2014 and April 2018 data were collected on n = 57,807 alcohol drinkers in England taking part in the Alcohol Toolkit Study (ATS). Respondents completed the AUDIT-C measure of frequency of alcohol consumption, amount consumed on a typical day and binge drinking frequency. The first two questions were used to derive a secondary measure of quantity: average weekly unit consumption. Socio-economic factors measured were: social-grade (based on occupation), employment status, educational qualifications, home and car ownership and income. Models were constructed using ridge regression to assess the contribution of each predictor taking account of high collinearity. Models were adjusted for age, gender and ethnicity.

**Results:**

The strongest predictor of frequency of alcohol consumption was social-grade. Those in the two lowest occupational categories of social grade (e.g. semi-skilled and unskilled manual workers, and unemployed, pensioners, casual workers) has fewer drinking occasions than those in professional-managerial occupations (β = -0.29, 95%CI -0.34 to -0.25; β = -0.31, 95%CI -0.33 to -0.29). The strongest predictor of consumed volume and binge drinking frequency was lower educational attainment: those whose highest qualification was an A-level (i.e. college/high school qualification) drank substantially more on a typical day (β = 0.28, 95%CI 0.25 to 0.31) and had a higher weekly unit intake (β = 3.55, 95%CI 3.04 to 4.05) than those with a university qualification. They also reported a higher frequency of binge drinking (β = 0.11, 95%CI 0.09 to 0.14). Housing tenure was a strong predictor of all drinking outcomes, while employment status and car ownership were the weakest predictors of most outcomes.

**Conclusion:**

Social-grade and educational attainment appear to be the strongest socioeconomic predictors of alcohol consumption indices in England, followed closely by housing tenure. Employment status and car ownership have the lowest predictive power.

## Introduction

In England, approximately 17% of adults drink at hazardous levels and around 1% can be classed as dependent [1]. However, there are substantial regional variations and a strong relationship with demographic characteristics, in particular, socio-economic status [2]. Numerous studies have indicated that people with higher socio-economic status tend to consume similar or greater amounts of alcohol than those of lower social-economic status, although the latter group seems to bear a disproportionate burden of negative alcohol-related consequences [3,4]. This phenomenon is known as the Alcohol Harm Paradox [5–7]. The complex relationship between socio-economic status and alcohol consumption may be partially driven by variations in drinking patterns [7], but also appears to be dependent on the specific measure of socio-economic status which is used. This is the first paper to our knowledge which has examined how far different measures of socio-economic status are associated with different alcohol consumption measures.

Using population level data we reported previously that whereas social-grade (an occupational based measure) has a U-shaped relationship with consumption, education has an inverse U-shaped relationship [8]. Other studies have also reported that higher educational attainment is associated with higher alcohol consumption [9] and that alcohol-related harm is disproportionately experienced by the most deprived in the lowest social-grade categorises [10,11]. There no longer appears to be an association with car ownership [8], which has been argued to be due to their increased affordability [12]. Studies have also failed to find associations with economic activity measured by employment status [13–15]. However, a strong association remains with another material indicator: housing tenure. A higher consumption but lower rate of binge drinking is generally found among those who own their own property [16]. The association with income and wealth is more complex. Despite more severe debt being associated with problem drinking [17], comparable consumption has been found across household income [4,14]. Although these differences offer some insight into what drives harmful alcohol consumption, they may also be reflect associations between other demographic characteristics (e.g. age, gender and ethnicity) and socio-economic measures [18].

The assessment of socio-economic status is a long-standing debate in the addictions field, given its multifaceted nature comprising of economic, social, educational and occupational factors [19]. Each measure has strengths and limitations. For example, income is affected by typically high non-response rates, reporting biases, monthly fluctuations and the fact that retained wealth is not captured [20]. The treatment of those still studying full time and of retirement age is problematic when looking at working status, and car ownership, which was once a strong predictor of health inequalities, no longer discriminates well between socio-economic groups [21]. The use of different measures across studies hinders comparisons and can often result in conflicting conclusions. A socio-economic measure highly predictive of one behaviour may also not be applicable to another. Although many have argued for the use of composite scores, these can involve increased cost and logistical constraints for survey designers as a result of increased survey length [22]. They can also present problems of interpretation and thereby create difficulties for policy development.

One problem in finding optimum measures for a given purpose is that different measures are typically highly correlated. The presence of multicollinearity means that it is difficult to identify those variables producing the largest associations with outcomes of interest using traditional statistical approaches. This is because the inclusion of collinear variables in the same model causes the variance of standard estimates to be inflated. A statistical technique that can overcome this is ridge regression. Ridge regression comes from the machine learning arena and can be seen as a penalised regression approach [23,24], which allows an assessment of the contribution of each independent variable while taking account of high collinearity.

Thus, this paper, applies ridge regression to assess the association between multiple measures of socio-economic status (i.e. income, home ownership, car ownership, education, employment status and social-grade; and a composite of these) with the three AUDIT-C measures: frequency of alcohol consumption, amount consumed on a typical drinking day and frequency of binge drinking, and an estimated mean weekly consumption derived from the AUDIT-QF [25]. Data are used from a large representative survey of adults aged 16+ and adjusted for gender, age and ethnicity. We are unaware of any study which has applied ridge regression to the problem of multicollinearity among socio-economic measures.

## Methods

### Ethical approval

Approval for the study was granted by UCL Ethics Committee (ID 0498/001). The data are collected by Ipsos Mori on behalf of UCL and are anonymised before being received by UCL. Explicit verbal agreement and willingness to answer questions voluntarily is recorded electronically by Ipsos Mori. This is standard protocol and was agreed by the UCL ethics committee. Participants are also given a printed information sheet.

### Design

Data were used from the ATS (www.alcoholinengland.info) between March 2014 and April 2018. The ATS involves monthly cross-sectional household computer-assisted interviews, conducted by Ipsos Mori of approximately 1,700 adults aged 16+ and over in England [26]. The baseline survey uses a type of random location sampling, which is a hybrid between random probability and simple quota sampling. England is first split into 171,356 ‘Output Areas’, comprising of approximately 300 households. These areas are then stratified based on ACORN characteristics and geographic region. ACORN is a socio-economic profiling tool developed by CACI (http://www.caci.co.uk/acorn/). The areas are then randomly allocated to interviewers, who travel to their selected areas and conduct the electronic interviews with one member of the household. Interviews are conducted until quotas based upon factors influencing the probability of being at home and tailored to local area census data are fulfilled. Morning interviews are avoided to maximise participant availability.

STROBE reporting guidelines are followed in this paper [27].

### Measures

Data were collected between March 2014 and April 2018 on participant’s age, gender, ethnicity, socio-economic status (SES) and drinking behaviour. Six measures of SES were collected which are outlined below.

1) Social-grade was measured using the British National Readership Survey (NRS) Social-Grade Classification Tool [28]: AB (Higher managerial, administrative or professional), C1 (Supervisory or clerical and junior managerial, administrative or professional), C2 (Skilled manual workers), D (Semi-skilled and unskilled manual workers) and E (Casual or lowest grade workers, pensioners, and others who depend on the welfare state for their income).

2) Gross annual household income in 15 bands (Up to £4499; £4,500–6,499; £6500–7499; £7500-£9499; £9500–11499; £11500-£13499; £13500–15499; £15500–17499; £17500–24999; £25000–29999; £30000–39999; £40000–49999; £50000–74999; £75000-£99999; > £100000).

3) Educational level in 8 categories (GCSE/O-level/CSE–high school sophomore; vocational qualification–high school senior; A-level or equivalent—high school senior; Bachelor degree or equivalent—university undergraduate; Masters/PhD or equivalent–university post-graduate; other; no formal qualifications–no post 16 qualifications; still studying)

4) Car ownership (owns a car; does not own a car)

5) Working status in 7 categories (Have paid job (full time); have a paid job (part time and over or under 8 hours per week); self-employed; full-time student; still at school; retired; not in paid work (long term illness, housewife or other reason)

6) Housing tenure in six categories (mortgage, owned outright, rented from local authority, rented from private landlord, belongs to housing association and other).

Due to violations of the assumption of linearity and in order to improve interpretation, all variables, except social-grade, were dichotomised or categorised as follows (all variables were coded so that lower SES or greater social-disadvantage reflects *higher* scores): 1) Income: four quartiles; 2) Education: University education, A-level and equivalent, GCSE/vocational, other/still studying and none; 3) Working status: Full time job versus no full-time job; and 4) Housing tenure: owner occupied (owned outright or being bought with a mortgage) versus other. These thresholds are based on previous research [8,29–31].

A composite score was also derived to assess how far this added predictive value over any one measure [19,22]. The composite score was coded such that a higher composite score reflected greater social disadvantage. The derived composite score was found to have good internal consistency (standardised Cronbach alpha of: 0.73).

Participants completed the AUDIT-C [32] which measures quantity of alcohol consumed on a typical day, frequency of alcohol consumption and binge drinking (i.e. single occasion high intensity consumption). It has been shown to be a sensitive and coherent measure of alcohol consumption [32,33]. An estimate of mean weekly unit consumption (one unit of alcohol is defined as 10 millilitres (8 grams) of pure alcohol and is a commonly used measure in the UK) was derived from the AUDIT-QF, which comprises of the first two questions of the AUDIT-C measuring quantity and frequency of alcohol consumption [25]. This was calculated by summing the scores for each item using the midpoint of the range in the response options, e.g. 2–3 drinking occasions per week meant 2.5. This AUDIT-QF derived weekly unit consumption measure has been used previously [34] and is in line with alternative measures not derived from AUDIT scores, including those used by the Sheffield Alcohol Policy Model (SAPM) [35]. The UK alcohol guidelines are also based on unit intake per week as opposed to on a typical drinking day [36].

### Analysis

The analysis plan was pre-registered on the Open Science Framework (https://osf.io/jub3q/). An amendment was made with an extension of data collection from March 2017 until April 2018. In the original protocol only the first three AUDIT-C questions were considered. It was decided after a discussion among the co-authors, that an estimate of weekly alcohol consumption should also be included. This was derived from the first two questions of the AUDIT (AUDIT-QF). Finally, it was decided to run two sensitivity analyses to check if different results were obtained for the linear regression using complete cases only for income and a missing data indicator. The large amount of missing data for income and use of the other SES variables for imputation may have artificially created stronger relationships between the variables and reduced the power of income in the models.

All analyses were conducted in R version 3.4.4. Prevalence of high-risk drinking was weighted using a rim (marginal) weighting technique. This involves an iterative sequence of weighting adjustments whereby separate nationally representative target profiles are set (for age, social grade, region, tenure, ethnicity, and working status within sex). This process is then repeated until all variables match the specified targets. Missing data were imputed by multiple imputation using the Amelia 11 package [37]. The number of imputed data sets were based on previous recommendations (i.e. n = 20) [38] and results combined using Rubin’s Rules [39]. The extent of missing data among the sample of drinkers was as follows: n = 14 (0.02%) for gender, n = 292 (0.51%) for age, n = 214 (0.37%) for ethnicity, n = 246 (0.42%) for car ownership, n = 390 (0.67%) for home ownership, n = 19,173 (33.2%) for income. An SES composite score, based on all six measures of SES, was derived from Multiple Correspondence Analysis (MCA) using the FactoMineR package [40]. Weights for the composite score comprised of those for the first three components; the assumption being that the variation explained by these is sufficient to adequately represent the original values [41]. The composite score was normalised to allow easier comparison with the dummy variables (i.e. it had a range of 0 to 1).

The analysis then proceeded as follows:

#### Association with individual socio-economic status measures

Separate linear models, specifying the Gaussian distribution family, were run to assess the associations between the socio-economic status measures and the four outcome measures of interest. Each model was reported unadjusted and adjusted for only age, gender and ethnicity.

#### Determining the best socio-economic status predictor

Model fit was compared using adjusted R-squared, AIC and BIC. Higher R-squared values and lower BIC and AIC values indicate a better model fit. Ten-fold cross validation was also performed to assess the predictive validity of each model [42]. Cross validation can be seen as a model validation technique for assessing how the results of a statistical analysis will generalize to an independent data set. Ten-fold cross-validation works by dividing the dataset into ten subsets. Each time, one of the k subsets is used as the test set and the other k-1 subsets are put together to form a training set. The training is then used to make predictions, and comparisons are made with the actual values in the test set. This gives what is known as the root-mean-square deviation (RMSE) which is the square root of the mean square error and reflects the differences between the actual response values and the predictions. Thus, lower values generally indicate a better prediction model.

To assess the predictive ability of each socio-economic variable when adjusting for all others, ridge regression was performed. The independent variables were too collinear to include in a typical multiple linear regression model. Multicollinearity occurs when highly correlated variables are simultaneously added to a regression model [43] and leads to biased standard errors and unstable p-values [43–45].

Ridge regression works by shrinking coefficients, with unimportant terms driven towards zero. The degree of penalisation, λ, is known as the ridge factor and must be estimated prior to data analysis. To choose λ, a cross validation approach was used whereby various models were fitted to the training set with different values of λ. The predictive accuracy of the models was then determined and the one which gave the most regularised simplest model chosen (where the cross-validated error was within one standard error of the model with minimum λ). It should be noted that this leads to coefficients which are slightly biased downwards but with the trade-off of much smaller standard errors and therefore large improvement in the precision of regression coefficients [24].

## Results

Between March 2014 and April 2018 data were collected on n = 57,807 (Prevalence: 68.3% 95CI 68.0 to 68.6) drinkers in England taking part in the Alcohol Toolkit Study (ATS). Descriptive statistics are given for the sample in Table 1.

**Table 1:** Mean (SD) frequency of consumption, quantity of alcohol consumption and binge drinking as frequency a function of socio-demographic characteristics (n = 57807).

		Frequency of alcohol consumption		Quantity of alcohol consumption		Binge drinking frequency		Weekly unit consumption	
	%(n)	M	SD	M	SD	M	SD	M	SD
**Overall**	100 (57807)	2.52	1.24	1.01	1.35	0.85	1.1	13.53	22.68
**Tenure**									
Owns home	67.6 (38087)	2.66	1.26	0.87	1.22	0.78	1.07	11.77	20.58
Does not own home	32.4 (18231)	2.23	1.16	1.3	1.56	1.01	1.13	17.21	26.16
**Employment**									
In full time work	48.0 (27054)	2.4	1.13	1.13	1.38	0.97	1.08	14.86	23.76
Not in full time work	52.0 (29264)	2.62	1.33	0.9	1.31	0.74	1.1	12.3	21.56
**Income**									
Quartile 1	16.0 (9036)	2.54	1.22	0.98	1.33	0.86	1.08	11.08	22.21
Quartile 2	22.3 (12542)	2.6	1.22	1.05	1.33	0.91	1.1	14.17	22.96
Quartile 3	13.7 (7720)	2.47	1.29	0.97	1.35	0.78	1.08	13.09	22.26
Quartile 4	13.9 (7847)	2.33	1.29	1.06	1.47	0.82	1.14	14.58	24.21
**Education**									
University	32.8 (18477)	2.7	1.21	0.83	1.15	0.84	1.05	11.11	19.43
A level & equivalent	19.9 (11193)	2.42	1.17	1.34	1.54	1.06	1.14	18.05	26.93
GCSE/vocational	27.3 (15394)	2.4	1.24	1.08	1.41	0.85	1.1	14.43	23.53
Other/still studying	7.6 (4290)	2.57	1.3	0.92	1.29	0.78	1.09	12.47	21.78
No post 16 qual	11.9 (6716)	2.4	1.35	0.83	1.31	0.61	1.08	11.4	20.6
**Car ownership**									
Owns car	13.6 (7642)	2.57	1.26	0.9	1.27	0.8	1.07	11.91	20.71
Does not own car	86.4 (48676)	2.51	1.24	1.02	1.36	0.86	1.1	13.79	22.97
**Social-grade**									
AB	28.5 (16066)	2.81	1.24	0.84	1.17	0.82	1.06	11.32	19.85
C1	33.9 (19109)	2.52	1.2	1.02	1.32	0.91	1.1	13.93	22.99
C2	19.1 (10780)	2.39	1.22	1.08	1.41	0.85	1.1	14.25	23.27
D	11.2 (6309)	2.19	1.21	1.1	1.48	0.81	1.1	14.89	24.39
E	7.2 (4054)	2.21	1.3	1.27	1.66	0.82	1.59	16.85	26.33
**Age**									
16–24	14.1 (7956)	2.12	0.98	1.77	1.68	1.31	1.1	23.22	30.46
25–34	13.1 (7358)	2.12	1.01	1.27	1.5	1.03	1.04	16.53	25.27
35–44	13.5 (7629)	2.39	1.11	1.07	1.33	0.96	1.07	14.09	22.58
45–54	16.1 (9093)	2.58	1.18	1.02	1.29	0.96	1.12	13.5	22.07
55–64	17.0 (9592)	2.76	1.28	0.87	1.19	0.79	1.13	11.72	20.27
65+	26.1 (14690)	2.8	1.43	0.51	0.92	0.44	0.94	7.71	14.97
**Gender**									
Male	53.4 (30051)	2.69	1.25	1.21	1.45	1.03	1.17	15.88	24.87
Female	44.6 (26267)	2.32	1.2	0.77	1.8	0.65	0.96	10.84	19.54
**Ethnicity**									
White	93.4 (52390)	2.55	1.25	1.02	1.34	0.87	1.1	13.76	22.9
Non-white	6.6 (3714)	2	1.08	0.76	1.2	0.64	0.94	10.32	19.13

### Association with individual socio-economic status measures

Table 2 shows results of the linear regression analyses assessing the association between socio-economic measures and the four outcome measures of interest before and after adjustment for age, gender and ethnicity. In general, those at greater social disadvantage reported consuming alcohol less frequently, but when they did they consumed larger amounts and were more likely to report ‘binge drinking’. There were a few exceptions, with those not in full time work on average having a higher frequency of consumption compared to those in full time work and those with GCSEs/vocational qualifications and with no qualifications less likely to report binge drinking relative to those with a university education. Those in social-grades C2 to E also reported less frequent binge drinking than those in social-grade AB. Table A in S1 File reports these results unadjusted for age, gender and ethnicity.

**Table 2:** Results of the adjusted linear and logistic regressions (for gender, age and ethnicity) assessing the association between individual measures of socio-economic status and frequency, quantity and binge drinking frequency (n = 57807).

	Frequency of alcohol consumption		Quantity of alcohol consumption		Binge drinking frequency		Weekly unit consumption	
	β_adjusted_	95%CI	β_adjusted_	95%CI	β_adjusted_	95%CI	β_adjusted_	95%CI
**Tenure**								
Owns home	Ref		Ref		Ref		Ref	
Does not own home	-0.21***	-0.23 to -0.19	0.17***	0.14 to 0.19	0.05***	0.03 to 0.07	2.20***	1.78 to 2.62
**Employment**								
In full time work	Ref		Ref		Ref		Ref	
Not in full time work	0.04**	0.01 to 0.06	0.13***	0.11 to 0.16	0.06***	0.04 to 0.08	1.72***	1.28 to 2.15
**Income**								
Quartile 1 (£50,000+)	Ref		Ref		Ref		Ref	
Quartile 2 (£25,000 to £49,999)	0.01	-0.02 to 0.03	0.11***	0.08 to 0.13	0.07***	-0.04 to 0.09	1.64***	1.17 to 2.10
Quartile 3 (£13,500 to £24,999)	-0.14***	-0.17 to -0.11	0.11***	0.07 to 0.14	0.01	-0.01 to 0.04	1.50***	0.95 to 2.06
Quartile 4 (up to £13,499)	-0.23***	-0.26 to -0.20	0.19***	0.15 to 0.22	0.06***	0.04 to 0.09	2.75***	2.20 to 3.30
**Education**								
University	Ref		Ref		Ref		Ref	
A level & equivalent	-0.20***	-0.23 to -0.17	0.30***	0.27 to 0.33	0.08***	0.06 to 0.11	4.21***	3.68 to 4.74
GCSE/vocational	-0.33***	-0.36 to -0.30	0.23***	0.20 to 0.26	-0.01	-0.03 to 0.01	2.92***	2.44 to 3.39
Other/still studying	-0.26***	-0.30 to -0.22	0.13***	0.08 to 0.17	-0.03	-0.06 to 0.01	1.71***	0.98 to 2.45
No post 16 qual	-0.52***	-0.56 to -0.49	0.25***	0.21 to 0.29	-0.04*	-0.07 to– 0.01	3.08***	2.45 to 3.71
Car ownership								
**Owns car**	Ref		Ref		Ref		Ref	
Does not own car	-0.03*	-0.06 to <-0.01	0.11***	0.08 to 0.14	0.06***	0.04 to 0.09	1.76***	1.23 to 2.29
**Social-grade**								
AB (highest)	Ref		Ref		Ref		Ref	
C1	-0.21***	-0.23 to -0.18	0.07***	0.39 to 0.10	0.02	<-0.01 to 0.04	1.10***	0.64 to 1.57
C2	-0.38***	-0.41 to -0.35	0.14***	0.11 to 0.17	-0.04**	-0.07 to -0.01	1.63***	1.10 to 2.17
D	-0.53***	-0.57 to -0.50	0.15***	0.101 to 0.18	<-0.01***	-0.11 to -0.05	2.12***	1.47 to 2.75
E (lowest)	-0.53***	-0.57 to -0.49	0.39***	0.35 to 0.44	-0.01	-0.03 to 0.04	5.02***	4.26 to 5.77
**Composite**	-0.09***	-0.10 to -0.08	0.13***	0.12 to 0.14	0.05***	0.04 to 0.06	1.81***	1.63 to 2.00
**Income (complete cases)**								
Quartile 1 (£50,000+)	Ref		Ref		Ref		Ref	
Quartile 2 (£25,000 to £49,999)	-0.26***	-0.29 to -0.22	0.06***	0.02 to 0.10	-0.12***	-0.15 to -0.09	0.73	-0.01 to 1.48
Quartile 3 (£13,500 to £24,999)	-0.41***	-0.45 to -0.37	0.06**	0.02 to 0.10	-0.17***	-0.21 to -0.14	1.49***	0.63 to 2.35
Quartile 4 (up to £13,499)	-0.51***	-0.55 to -0.47	0.15***	0.11 to 0.19	-0.13***	-0.17 to -0.10	2.35***	1.48 to 3.22
**Income (Missing data indicator)**								
Quartile 1 (£50,000+)	Ref		Ref		Ref		Ref	
Quartile 2 (£25,000 to £49,999)	-0.25	-0.28 to -0.21	0.05**	0.02 to 0.09	-0.12***	-0.15 to -0.09	0.62	-0.10 to 1.36
Quartile 3 (£13,500 to £24,999)	-0.39***	-0.43 to -0.35	0.05*	0.01 to 0.09	-0.18***	-0.21 to -0.14	1.26**	0.44 to 2.09
Quartile 4 (up to £13,499)	-0.48***	-0.52 to -0.44	0.13***	0.09 to 0.17	-0.14***	-0.17 to -0.10	2.02***	1.19 to 2.86
Missing	-0.35***	-0.38 to -0.32	-0.05**	-0.08 to -0.01	-0.25***	-0.28 to -0.23	-0.94**	-1.64 to -0.25

Note

* significant at p<0.05

** significant at p<0.01

*** significant at p<0.001

Standardised coefficients are given for the composite score (mean 0.51 and SD 0.20)

### Determining the best socio-economic status predictor–linear regression

Table 3 and Table B in S1 File give the fit indices and RMSE from the 10-fold cross-validation for the models reported in Table 2 and Table A in S1 File. These suggest that the best predictor of frequency of consumption is social-grade and the composite score is the best predictor of quantity of alcohol consumed, frequency of binge drinking and mean average weekly unit consumption. Educational qualifications appeared to be the next best individual predictor across the outcome measures and housing tenure also performed well.

**Table 3:** Model fit statistics (R-squared, AIC and BIC) and mean squared prediction error from 10-fold cross validation for the regression models presented in Table 2 (n = 57807).

	Adjusted Model		
	R^2^*100	AIC/BIC	RMSE [61]
Tenure ~ frequency	8.141	179534.8/179624.2	1.191
Employment ~ frequency	7.630	179847.2/179936.6	1.194
Income ~ frequency	8.084	179571.6/179678.9	1.192
Income* ~ frequency	8.566	-—-—-—-—-—-—- -	1.214
Income^#^ ~ frequency	7.937	180280.5/180396.5	1.227
Education ~ frequency	9.474	178714.6/178830.8	1.183
Car ownership ~ frequency	7.621	179852.6/179941.9	1.195
Social grade ~ frequency	**9.923**	**178434.9/178551.1**	**1.180**
Composite ~ frequency	8.088	179567.5/179656.9	1.192
Tenure ~ quantity	12.725	186036.3/186125.7	1.262
Employment ~ quantity	12.604	186114.8/186204.2	1.263
Income ~ quantity	12.694	186058.1/186165.4	1.262
Income* ~ quantity	11.108	-—-—-—-—-—-—- -	1.298
Income^#^ ~ quantity	12.188	186524.9/186640.9	1.306
Education ~ quantity	13.197	185733.9/185850.1	1.259
Car ownership ~ quantity	12.518	186170.3/186259.6	1.263
Social grade ~ quantity	12.967	185883.4/185999.6	1.260
Composite ~ quantity	**13.252**	**185695.5/185784.9**	**1.258**
Tenure ~ binge drinking	10.967	163558.3/163647.7	1.034
Employment ~ binge drinking	10.988	163545.1/163634.5	1.034
Income ~ binge drinking	10.998	162541.0/163648.3	1.033
Income* ~ binge drinking	10.840	-—-—-—-—-—-—- -	1.054
Income^#^ ~ quantity			
Education ~ binge drinking	11.042	163513.8/163630.0	1.033
Car ownership ~ binge drinking	10.965	163559.5/163648.9	1.034
Social grade ~ binge drinking	11.019	163528.3/163644.5	1.033
Composite ~ binge drinking	**11.094**	**163477.8/163567.2**	**1.033**
Tenure ~ weekly units	6.635	507573.8/507633.2	21.918
Employment ~ weekly units	6.563	507617.2/507706.6	21.926
Income ~ weekly units	6.661	507559.9/507667.2	21.912
Income* ~ weekly units	4.602	-—-—-—-—-—-—- -	27.194
Income^#^ ~ quantity	5.217	519658.3/519774.3	26.543
Education ~ weekly units	6.949	507387.3/507503.5	21.882
Car ownership ~ weekly units	6.533	507635.3/507724.5	21.930
Social grade ~ weekly units	6.762	507500.0/507616.2	21.903
Composite ~ weekly units	**7.056**	**507319.3/507408.7**	**21.863**

* Sensitivity analysis: complete cases only for income

^#^ Sensitivity analysis: missing data indicator; AIC and BIC are not given for the complete case analysis as they both depend on sample size and can therefore not be compared to the other results where sample size is larger due to multiple imputation of missing values; adjusted R^2^ corrects for differences in sample size; RMSE is more valid for the main analysis due to the larger sample size.

### Determining the best socio-economic status predictor–ridge regression

Table 4 reports the results from the *best* ridge regression models adjusted for gender, age and ethnicity, and all measures of SES. File A in the S1 File and Figures A and B in S1 File describe the ridge regression models at different values of λ. The strongest predictor of frequency of alcohol consumption was social-grade. The strongest predictor of quantity of consumption, binge drinking frequency and weekly unit consumption, was educational attainment. Housing tenure was also a consistently strong predictor across all outcome measures. Educational qualification also acted as a good predictor of frequency of alcohol consumption and social-grade as a good predictor of quantity of alcohol consumption and weekly unit intake. Car ownership and employment status were generally the poorest predictors, while income had some predictive value particularly in the comparison of the highest and lowest earners. Table C in S1 File reports the results from the best ridge models adjusted for all measures of SES but with no adjustment for gender, age and ethnicity.

**Table 4:** Results of the ridge regression at optimal values of lambda (adjusted for sex, age and ethnicity, and all measures of SES) (n = 57807).

	Frequency of alcohol consumption		Quantity of alcohol consumption		Binge drinking frequency		Weekly unit consumption	
	β	95%CI	β	95%CI	β	95%CI	β	95%CI
**Tenure**								
Owns home	Ref		Ref		Ref		Ref	
Does not own home	-0.13*	-0.15 to -0.01	0.16*	0.14 to 0.19	0.10*	0.08 to 0.13	2.31*	1.84 to 2.78
**Employment**								
In full time work	Ref	Ref	Ref		Ref		Ref	
Not in full time work	0.14*	0.11 to 0.16	<0.01	-0.03 to 0.02	<0.01	-0.02 to 0.02	-0.39	-0.86 to 0.08
**Income**								
Quartile 1 (£50,000+)	Ref	Ref	Ref		Ref		Ref	
Quartile 2 (£25,000 to £49,999)	0.02	-0.01 to 0.05	0.07*	0.04 to 0.01	0.04*	0.01 to 0.06	0.80*	0.23 to 1.30
Quartile 3 (£13,500 to £24,999)	-0.05*	-0.08 to -0.02	0.02	-0.01 to 0.06	-0.02	-0.04 to 0.01	0.14	-0.45 to 0.73
Quartile 4 (up to £13,499)	-0.10*	-0.13 to -0.07	0.05*	0.02 to 0.08	0.03*	<0.01 to 0.05	0.86*	0.34 to 1.37
**Education**								
University	Ref		Ref		Ref		Ref	
A level and equivalent	-0.11*	-0.14 to -0.08	0.28*	0.25 to 0.31	0.11*	0.09 to 0.14	3.55*	3.04 to 4.05
GCSE/vocational	-0.16*	-0.19 to -0.12	0.15*	0.11 to 0.19	<0.01	-0.03 to 0.04	1.44*	0.70 to 2.18
Other/still studying	-0.10*	-0.14 to -0.06	0.06*	0.02 to 0.10	-0.02	-0.05 to 0.01	0.41	-0.3 to 1.12
No post 16 qual	-0.22*	-0.25 to -0.19	0.07*	0.04 to 0.10	-0.06*	-0.09 to -0.04	0.07	-0.45 to 0.59
**Car ownership**								
Owns car	Ref		Ref		Ref		Ref	
Does not own car	-0.02	-0.05 to 0.01	0.09*	0.06 to 0.11	0.05*	0.02 to 0.07	1.24*	0.76 to 1.71
**Social-grade**								
AB	Ref		Ref		Ref		Ref	
C1	-0.09*	-0.13 to -0.06	0.01	-0.02 to 0.05	0.02*	<0.01 to 0.05	0.38	-0.2 to 0.96
C2	-0.19*	-0.23 to -0.15	0.05*	0.01 to 0.09	-0.03	-0.06 to <0.01	0.51	-0.2 to 1.22
D	-0.29*	-0.34 to -0.25	0.05*	<0.01 to 0.09	-0.07*	-0.11 to -0.03	0.86*	0.01 to 1.71
E	-0.31*	-0.33 to -0.29	0.24*	0.22 to 0.26	-0.02	-0.04 to <0.01	2.74*	2.37 to 3.10

**Note:** standard errors for ridge regression are biased to allow accurate estimation of coefficients

* significant at p<0.05.

### Sensitivity analysis–complete case analysis for income

The results of the sensitivity analysis are presented in Tables 2 and 3. This shows that after only choosing complete cases and including a missing data indicator that income generally remained a poorer predictor of the outcome measures of interest relative to social-grade, tenure and educational achievements. Of interest, is that those with missing data self-reported a lower frequency and quantity of alcohol consumption and less frequent binge drinking compared to those earning in the upper quartile.

## Discussion

In the linear regression analysis, the composite score was found to outperform all six individual SES measures except in the case of frequency of consumption, where social-grade appeared to offer the best predictive power. In the ridge regression analysis, the strongest predictor of frequency of alcohol consumption was social-grade, while the strongest predictor of quantity of consumption and binge drinking frequency was educational attainment. Housing tenure was also a consistently strong predictor while car ownership and employment status were poor predictors of most outcomes. Income offered some predictive value.

It is unsurprising that the composite measure outperformed the individual measures of SES [21] in the linear regression analysis but it has disadvantages. Using a composite measure of individual level variables may obscure the underlying mechanisms, as evident by differences in the associations reported here, and prevent understanding of how different aspects of SES contribute to alcohol use. Composite scores also come at greater cost, both financial and logistical in terms of respondent time and number of survey items. Thus, they are not always suitable for survey-based studies. There was one exception: frequency of consumption was better predicted by social-grade, an occupation-based classification system. The social-grade A:E measure has several advantages including its wide use across surveys both in the UK and Europe, allowing for easy comparison, but can be time consuming in itself [46].

The ridge regression analysis allowed assessment of the specific contribution of each SES predictor while taking account of high collinearity between these predictors. Educational qualification emerged as the best predictor of consumption on a typical drinking day, weekly unit consumption and binge drinking frequency in the ridge regression analysis. The strongest predictor of frequency of alcohol consumption remained social-grade, but this was closely followed by educational attainments. Previous studies have also reported that higher educational attainment is associated with higher alcohol consumption [8,9]. Previous studies have shown strong associations between level of education and alcohol abuse and dependence in later life [47,48], and several possible explanations can be given for this association. The ‘human capital’ approach would argue that education increases individuals’ ability to synthesise information on the health implications of alcohol use or that those with greater educational qualifications have more health-orientated allocation of resources [49]. It may also be that there is no causal association but that future-orientated individuals invest more in their health and are more educated [49]. Alternatively, more educated individuals may prefer healthy habits and avoid unhealthy ones and education is a key component of health literacy [50,51]. Finally, more educated individuals may have more material resources which can help buffer adverse effects of drinking by better nutrition or living in places with less social harm [5,6]. It will be important to try and disentangle what may be driving the association as this could have significant policy implications, including perhaps the targeting of interventions to those without post-16 qualifications. If the association is causal, this also strengthens the economic case for providing free, high quality post-16 education to everyone. It is also of interest that housing tenure was a consistently strong predictor across all outcome measures while car ownership and employment status were poorer predictors. Previous studies have similarly found housing tenure to be strongly related with heavy intake and problem drinking [52]. There are several possible explanations for this, including the local environment and culture of ‘owned’ homes relative to rented and social housing [53]. Those in social housing also often experience greater levels of depression and poor mental health which themselves are associated with heavy drinking patterns [54]. Previously it had been thought that car ownership was an indicator of affluence due to the costs associated with purchase and maintenance; however, questions have been raised whether it is still an appropriate measure[53], with 75% of households having access to a car and 42.6% multiple vehicles [12].

Household income offered some predictive power and unlike education and occupation measures, gives a good indication of the standard of living and life chances of a household. However, questions regarding personal income are often met with hostility as evident by the large amounts of missing data in the current study. Household members may also not have equal access to the income which blurs the association with alcohol use [55]. This may explain why previous studies have found that the association between alcohol consumption and individual wealth is complex [4,14,17].

These findings have several implications. First, the finding that income, although not the best predictor of alcohol consumption, was still significantly associated supports previous arguments that those of lower socio-economic status have more to gain from the most effective public health alcohol policies–namely, increasing taxation and setting a minimum unit price [56]. Secondly, they provide guidance as to which measures one may wish to use when identifying individuals most at risk from harmful alcohol intake. In effect, this can help to tailor interventions and supports the concept of personalised medicine [57]. Thirdly, although these findings suggest that ideally multiple measures of socio-economic status are used in population surveys, they offer some guidance as to which socio-economic measures to choose when there are financial or logistical constraints and the goal is to assess associations with alcohol frequency and quantity. Finally, the differing associations with frequency of consumption and amount consumed may help to partially explain the AHP [6]. Although those of a higher social-grade consume alcohol more frequently, those with fewer educational attainments drink larger quantities and this may drive the higher rates of alcohol-related harm that lower SES groups experience. Previous studies have shown that lower SES groups are more likely to drink at extreme levels [7].

This study has several advantages including the use of data from a large household survey of adults in England and the widely validated AUDIT questionnaire [32]. However, this study also has several limitations which must be considered. As with all cross-sectional surveys, caution should be taken when assigning cause and effect. It may be the case that SES has a direct influence on drinking behaviour but drinking behaviour may also have an effect on some of the SES measures. For example, those who experience greater alcohol problems may be more likely to become unemployed. Self-report measures are also susceptible to recall bias. Secondly, although this paper assessed a wide range of SES measures which reflect those used previously; the measures did not address the social capital aspect of SES. This is something which may require further consideration, as family and friend networks are associated with health outcomes [58]. Thirdly, despite ridge regression being recommended for multicollinearity problems [59], some have raised concerns about the use of biased regression methods to assign relative importance to independent variables in the presence of multicollinearity [13,60]. Although such concerns should be noted when drawing conclusions, the consistency between the results from the ridge regression and linear regression models gives some validity to the conclusions drawn here. Fourthly, although we adjusted for several demographic characteristics some of these findings may be accounted for by other factors which are correlated with SES, including area level deprivation and marital status. These will be important factors to consider in future research. Fifthly, while the sample was designed to be representative, there is a risk of bias in terms of the characteristics of those who agree to participate. There is also a risk that respondents may underestimate or fail to report their drinking. As with all population level surveys, interviewer effects are also possible whereby answers are affected by the interviewer administering the survey. Finally, this study assessed how socio-economic measures are associated with alcohol consumption but not why they are. Additional qualitative and longitudinal research is needed to address this question. Part of the explanation relates to how the socio-economic measures assess somewhat different (albeit related) constructs, rather than simply being better or worse assessments of socio-economic position.

In conclusion, educational achievements appear to be the best predictors of alcohol use, both measures of frequency and amount consumed, followed closely by social-grade and housing tenure. Employment status and car ownership have less predictive power.

## Supporting information

S1 File *Table A* gives the results on the unadjusted linear regressions assessing the association between individual measures of socio-economic status and frequency, quantity and binge drinking. *Table B* gives the model fit statistics and mean squared error from 10-fold cross validation for the regression models presented in *Table A*. *Table C* gives the results of the ridge regression at optimal values of lambda unadjusted for sex, age and ethnicity. *File A* gives information on how the best ridge regression model was chosen. Figure A gives the results of the ridge regression at different values of Log(Lambda) for predicting a) frequency, b) quantity and c) frequency of binge drinking (unadjusted). Figure B gives the results of the ridge regression at different values of Log(Lambda) for predicting a) frequency, b) quantity and c) frequency of binge drinking (adjusted).
(DOCX)

## References

[1] McManus S BP, Jenkins R, Brugha T (2016) Adult Psychiatric Morbidity Survey: Survey of Mental Health and Wellbeing, England, 2014 Leeds.

[2] Beard E, Brown J, West R, Angus C, Kaner E, et al (2017) Healthier central England or North–South divide? Analysis of national survey data on smoking and high-risk drinking. BMJ open 7: e014210 10.1136/bmjopen-2016-014210 28249851 PMC5353327

[3] Collins SE (2016) Associations Between Socioeconomic Factors and Alcohol Outcomes. Alcohol research: current reviews 38: 83–94. 27159815 10.35946/arcr.v38.1.11 PMC4872618

[4] Katikireddi SV, Whitley E, Lewsey J, Gray L, Leyland AH (2017) Socioeconomic status as an effect modifier of alcohol consumption and harm: analysis of linked cohort data. The Lancet Public Health 2: e267–e276. 10.1016/S2468-2667(17)30078-6 28626829 PMC5463030

[5] Bellis MA, Hughes K, Nicholls J, Sheron N, Gilmore I, et al (2016) The alcohol harm paradox: using a national survey to explore how alcohol may disproportionately impact health in deprived individuals. BMC public health 16: 111 10.1186/s12889-016-2766-x 26888538 PMC4758164

[6] Bellis M, Jones L, Morleo M (2015) Understanding the alcohol harm paradox. Alcohol Research UK Conference, 2013.

[7] Lewer D, Meier P, Beard E, Boniface S, Kaner E (2016) Unravelling the alcohol harm paradox: a population-based study of social gradients across very heavy drinking thresholds. BMC public health 16: 599 10.1186/s12889-016-3265-9 27430342 PMC4950253

[8] Beard E, Brown J, West R, Angus C, Brennan A, et al (2016) Deconstructing the Alcohol Harm Paradox: A population based survey of adults in England. PloS one 11: e0160666 10.1371/journal.pone.0160666 27682619 PMC5040414

[9] Smith GW, Shevlin M, Murphy J, Houston JE (2010) An assessment of the demographic and clinical correlates of the dimensions of alcohol use behaviour. Alcohol and alcoholism 45: 563–572. 10.1093/alcalc/agq052 20876219

[10] Erskine S, Maheswaran R, Pearson T, Gleeson D (2010) Socioeconomic deprivation, urban-rural location and alcohol-related mortality in England and Wales. BMC Public Health 10: 99 10.1186/1471-2458-10-99 20184763 PMC2841677

[11] Ally AK, Lovatt M, Meier PS, Brennan A, Holmes J (2016) Developing a social practice‐based typology of British drinking culture in 2009–2011: implications for alcohol policy analysis. Addiction 111: 1568–1579. 10.1111/add.13397 27095617 PMC5094536

[12] Lansley G (2016) Cars and socio-economics: understanding neighbourhood variations in car characteristics from administrative data. Regional Studies, Regional Science 3: 264–285.

[13] Smith G, Campbell F (1980) A critique of some ridge regression methods. Journal of the American Statistical Association 75: 74–81.

[14] Seid AK, Bloomfield K, Hesse M (2018) The relationship between socioeconomic status and risky drinking in Denmark: a cross-sectional general population study. BMC public health 18: 743 10.1186/s12889-018-5481-y 29907145 PMC6003187

[15] Feng W, Zhou W, Butler JS, Booth BM, French MT (2001) The impact of problem drinking on employment. Health Econ 10: 509–521. 11550292 10.1002/hec.611

[16] Fone DL, Farewell DM, White J, Lyons RA, Dunstan FD (2013) Socioeconomic patterning of excess alcohol consumption and binge drinking: a cross-sectional study of multilevel associations with neighbourhood deprivation. BMJ Open 3. 10.1136/bmjopen-2012-002337 PMC3641461 23587771

[17] Richardson T, Elliott P, Roberts R (2013) The relationship between personal unsecured debt and mental and physical health: a systematic review and meta-analysis. Clin Psychol Rev 33: 1148–1162. 10.1016/j.cpr.2013.08.009 24121465

[18] Obradors-Rial N, Ariza C, Rajmil L, Muntaner C (2018) Socioeconomic position and occupational social class and their association with risky alcohol consumption among adolescents. International Journal of Public Health 63: 457–467. 10.1007/s00038-018-1078-6 29396604

[19] Batista-Foguet J, Fortiana J, Currie C, Villalbi J (2004) Socio-economic indexes in surveys for comparisons between countries. Social Indicators Research 67: 315–332.

[20] Wagstaff A, O'Donnell O, Van Doorslaer E, Lindelow M (2007) Analyzing health equity using household survey data: a guide to techniques and their implementation: World Bank Publications.

[21] Galobardes B, Lynch J, Smith GD (2007) Measuring socioeconomic position in health research. British medical bulletin 81: 21 10.1093/bmb/ldm001 17284541

[22] Organisation for Economic Co-operation and Development (2008) Handbook on constructing composite indicators: Methodology and user guide Paris: OECD Publishing.

[23] Hoerl AE, Kennard RW (1970) Ridge regression: applications to nonorthogonal problems. Technometrics 12: 69–82.

[24] Cule E, De Iorio M (2013) Ridge regression in prediction problems: automatic choice of the ridge parameter. Genetic epidemiology 37: 704–714. 10.1002/gepi.21750 23893343 PMC4377081

[25] Aalto M, Tuunanen M, Sillanaukee P, Seppä K (2006) Effectiveness of Structured Questionnaires for Screening Heavy Drinking in Middle‐Aged Women. Alcoholism: Clinical and Experimental Research 30: 1884–1888. 10.1111/j.1530-0277.2006.00233.x 17067353

[26] Beard E, Brown J, West R, Acton C, Brennan A, et al (2015) Protocol for a national monthly survey of alcohol use in England with 6-month follow-up:‘The Alcohol Toolkit Study’. BMC public health 15: 230 10.1186/s12889-015-1542-7 25884652 PMC4363185

[27] Von Elm E, Altman DG, Egger M, Pocock SJ, Gøtzsche PC, et al (2007) The Strengthening the Reporting of Observational Studies in Epidemiology (STROBE) statement: guidelines for reporting observational studies. Preventive medicine 45: 247–251. 10.1016/j.ypmed.2007.08.012 17950122

[28] Collis D (2009) Social grade: A classification tool–Bite sized through piece.

[29] National Statistics Department for Work and Pensions (2011) Households Below Average Income: An Analysis of the Income Distribution 1994/95-2009/10.

[30] Eachus J, Chan P, Pearson N, Propper C, Smith GD (1999) An additional dimension to health inequalities: disease severity and socioeconomic position. Journal of Epidemiology and Community Health 53: 603–611. 10616672 10.1136/jech.53.10.603 PMC1756792

[31] Galobardes B, Shaw M, Lawlor DA, Lynch JW, Smith GD (2006) Indicators of socioeconomic position (part 1). Journal of epidemiology and community health 60: 7–12. 10.1136/jech.2004.023531 PMC2465546 16361448

[32] Saunders JB, Aasland OG, Babor TF, de la Fuente JR, Grant M (1993) Development of the Alcohol Use Disorders Identification Test (AUDIT): WHO Collaborative Project on Early Detection of Persons with Harmful Alcohol Consumption—II. Addiction 88: 791–804. 8329970 10.1111/j.1360-0443.1993.tb02093.x

[33] Frank D, DeBenedetti AF, Volk RJ, Williams EC, Kivlahan DR, et al (2008) Effectiveness of the AUDIT-C as a Screening Test for Alcohol Misuse in Three Race/Ethnic Groups. Journal of General Internal Medicine 23: 781–787. 10.1007/s11606-008-0594-0 18421511 PMC2517893

[34] Lima CT, Freire ACC, Silva APB, Teixeira RM, Farrell M, et al (2005) Concurrent and construct validity of the AUDIT in an urban Brazilian sample. Alcohol and Alcoholism 40: 584–589. 10.1093/alcalc/agh202 16143704

[35] Brennan A, Meier P, Purshouse R, Rafia R, Meng Y, et al (2015) The Sheffield alcohol policy model–a mathematical description. Health economics 24: 1368–1388. 10.1002/hec.3105 25270223

[36] Buykx P, Li J, Gavens L, Hooper L, Gomes de Matos E, et al (2018) Self-reported knowledge, correct knowledge and use of UK drinking guidelines among a representative sample of the English population. Alcohol and alcoholism 53: 453–460. 10.1093/alcalc/agx127 29351574 PMC6016612

[37] Honaker J, King G, Blackwell M, Blackwell MM (2010) Package ‘Amelia’. Version.

[38] Graham JW, Olchowski AE, Gilreath TD (2007) How many imputations are really needed? Some practical clarifications of multiple imputation theory. Prevention Science 8: 206–213. 10.1007/s11121-007-0070-9 17549635

[39] Rubin DB (1996) Multiple imputation after 18+ years. Journal of the American Statistical Association 91: 473–489.

[40] Husson F, Josse J, Le S, Mazet J, Husson MF (2014) Package ‘FactoMineR’.

[41] Ram R (1982) Composite indices of physical quality of life, basic needs fulfilment, and income: A ‘principal component’representation. Journal of Development Economics 11: 227–247.

[42] Kohavi R. A study of cross-validation and bootstrap for accuracy estimation and model selection; 1995 Stanford, CA. pp. 1137–1145.

[43] Alin A (2010) Multicollinearity. Wiley Interdisciplinary Reviews: Computational Statistics 2: 370–374.

[44] Farrar DE, Glauber RR (1967) Multicollinearity in regression analysis: the problem revisited. The Review of Economic and Statistics: 92–107.

[45] Wang GC (1996) How to handle multicollinearity in regression modeling. The Journal of Business Forecasting 15: 23.

[46] Bradley N (2007) Marketing research: tools & techniques: Oxford University Press, USA.

[47] Crum RM, Helzer JE, Anthony JC (1993) Level of education and alcohol abuse and dependence in adulthood: a further inquiry. Am J Public Health 83: 830–837. 8498620 10.2105/ajph.83.6.830 PMC1694736

[48] Iparraguirre J (2015) Socioeconomic determinants of risk of harmful alcohol drinking among people aged 50 or over in England. BMJ Open 5. 10.1136/bmjopen-2015-007684 PMC4521517 26204909

[49] Grossman M (2010) The relationship between health and schooling. Investing In Human Capital For Economic Development In China: World Scientific. pp. 279–291.

[50] Rahkonen O, Berg M-A, Puska P (1995) Relationship between educational status, gender and smoking in Finland, 1978–1992. Health Promotion International 10: 115–120.

[51] Zarcadoolas C, Pleasant A, Greer DS (2005) Understanding health literacy: an expanded model. Health promotion international 20: 195–203. 10.1093/heapro/dah609 15788526

[52] Batty GD, Lewars H, Emslie C, Benzeval M, Hunt K (2008) Problem drinking and exceeding guidelines for 'sensible' alcohol consumption in Scottish men: associations with life course socioeconomic disadvantage in a population-based cohort study. BMC Public Health 8: 302 10.1186/1471-2458-8-302 18761741 PMC2538536

[53] Ellaway A, Macdonald L, Kearns A (2016) Are housing tenure and car access still associated with health? A repeat cross-sectional study of UK adults over a 13-year period. BMJ Open 6: e012268 10.1136/bmjopen-2016-012268 27807086 PMC5128997

[54] Ellaway A, McKay L, Macintyre S, Kearns A, Hiscock R (2004) Are social comparisons of homes and cars related to psychosocial health? International Journal of Epidemiology 33: 1065–1071. 10.1093/ije/dyh197 15256528

[55] Duncan GJ, Daly MC, McDonough P, Williams DR (2002) Optimal indicators of socioeconomic status for health research. American journal of public health 92: 1151–1157. 12084700 10.2105/ajph.92.7.1151 PMC1447206

[56] Holmes J, Meng Y, Meier PS, Brennan A, Angus C, et al (2014) Effects of minimum unit pricing for alcohol on different income and socioeconomic groups: a modelling study. The Lancet 383: 1655–1664. 10.1016/S0140-6736(13)62417-4 PMC4018486 24522180

[57] Vogenberg FR, Isaacson Barash C, Pursel M (2010) Personalized Medicine: Part 1: Evolution and Development into Theranostics. Pharmacy and Therapeutics 35: 560–576. 21037908 PMC2957753

[58] Berkman LF, Glass T (2000) Social integration, social networks, social support, and health. Social epidemiology 1: 137–173.

[59] Duzan H, Shariff NSBM (2015) Ridge regression for solving the multicollinearity problem: review of methods and models. Journal of Applied Sciences 15: 392.

[60] Pasha G, Shah M (2004) Application of ridge regression to multicollinear data. Journal of research (Science) 15: 97–106.

[61] Algina J, Keselman H (2000) Cross-validation sample sizes. Applied Psychological Measurement 24: 173–179.

