# Correction: Associations between socio-economic factors and alcohol consumption: A population survey of adults in England

**DOI:** 10.1371/journal.pone.0216378

**Published:** 2019-04-30

**Authors:** Emma Beard, Jamie Brown, Robert West, Eileen Kaner, Petra Meier, Sadie Boniface, Susan Michie

Dr. Sadie Boniface should be included in the author byline. They should be listed as the sixth author and affiliated with Addictions Department, Kings College London, London, UK. The contributions of this author are as follows: Writing—review & editing.

The correct citation is: Beard E, Brown J, West R, Kaner E, Meier P, Boniface S (2019) Associations between socio-economic factors and alcohol consumption: A population survey of adults in England. PLoS ONE 14(2): e0209442. https://doi.org/10.1371/journal.pone.0209442
