# Supplementary material for: Associations between socio-economic factors and alcohol consumption: A population survey of adults in England
Source: PLoS One. 2019 Feb 4;14(2):e0209442. doi: 10.1371/journal.pone.0209442 (PMC6361426; doi:10.1371/journal.pone.0209442)
Supplement: S1 File — Table A gives the results on the unadjusted linear regressions assessing the association between individual measures of socio-economic status and frequency, quantity and binge drinking. Table B gives the model fit statistics and mean squared error from 10-fold cross validation for the regression models presented in Table A. Table C gives the results of the ridge regression at optimal values of lambda unadjusted for sex, age and ethnicity. File A gives information on how the best ridge regression model was chosen. Figure A gives the results of the ridge regression at different values of Log(Lambda) for predicting a) frequency, b) quantity and c) frequency of binge drinking (unadjusted). Figure B gives the results of the ridge regression at different values of Log(Lambda) for predicting a) frequency, b) quantity and c) frequency of binge drinking (adjusted). (DOCX) [file pone.0209442.s001.docx]

**Supplementary Table A:** Results of the unadjusted linear regressions assessing the association between individual measures of socio-economic status and frequency, quantity and binge drinking frequency

|  | Frequency of alcohol consumption | | Quantity of alcohol consumption | | Binge drinking frequency | | Weekly unit consumption | |
| --- | --- | --- | --- | --- | --- | --- | --- | --- |
|  | Β_unadjusted_ | 95%CI | Β_unadjusted_ | 95%CI | Β_unadjusted_ | 95%CI | Β_unadjusted_ | 95%CI |
| **Tenure**  Owns home  Does not own home | Ref  -0.43*** | -0.45 to -0.41 | Ref  0.44*** | 0.41 to 0.46 | Ref  0.23*** | 0.21 to 0.25 | Ref  5.44*** | 5.04 to 5.84 |
| **Employment**  In full time work  Not in full time work | Ref  0.22*** | 0.20 to 0.24 | Ref  -0.23*** | -0.25 to -0.21 | Ref  -0.23*** | -0.25 to -0.21 | Ref  -2.56*** | -2.94 to -2.19 |
| **Income**  Quartile 1 (£50,000+)  Quartile 2 (£25,000 to £49,999)  Quartile 3 (£13,500 to £24,999)  Quartile 4 (up to £13,499) | Ref  0.06***  -0.07***  -0.21*** | 0.03 to 0.08  -0.10 to -0.04  -0.24 to -0.18 | Ref  0.07***  -0.02  0.08*** | 0.04 to 0.10  -0.05 to 0.02  0.04 to 0.11 | Ref  0.6***  -0.08***  -0.04*** | 0.03 to 0.07  -0.11 to -0.05  -0.07 to -0.01 | Ref  1.09***  0.02  1.52*** | 0.61 to 1.57  -0.56 to 0.59  0.93 to 2.07 |
| **Education**  University  A level & equivalent  GCSE/vocational  Other/still studying  No post 16 qual | Ref  -0.28***  -0.30***  -0.13***  -0.31*** | -0.31 to -0.26  -0.33 to -0.27  -0.17 to -0.09  -0.34 to -0.27 | Ref  0.52***  0.26***  0.09***  0.01 | 0.48 to 0.55  0.23 to 0.29  0.05 to 0.13  -0.03 to 0.05 | Ref  0.22***  0.01  -0.06**  -0.23*** | 0.19 to 0.24  -0.02 to 0.03  -0.10 to -0.02  -0.26 to -0.20 | Ref  6.94***  3.32***  1.37***  0.30 | 6.41 to 7.47  2.84 to 3.81  0.61 to 2.11  -0.33 to 0.93 |
| **Car ownership**  Owns car  Does not own car | Ref  -0.06*** | -0.09 to -0.03 | Ref  0.12*** | 0.09 to 0.15 | Ref  0.06*** | 0.03 to 0.09 | Ref  1.87*** | 1.33 to 2.42 |
| **Social-grade**  AB (highest)  C1  C2  D  E (lowest) | Ref  -0.30***  -0.42***  -0.63***  -0.61*** | -0.32 to -0.27  -0.45 to -0.39  -0.66 to -0.59  -0.65 to -0.57 | Ref  0.18***  0.24***  0.26***  0.43*** | 0.15 to 0.20  0.21 to 0.28  0.23 to 0.30  0.38 to 0.47 | Ref  0.09***  0.03*  -0.08  <0.01 | 0.07 to 0.11  0.01 to 0.06  -0.05 to 0.02  -0.04 to 0.04 | Ref  2.51***  2.93***  3.57***  5.53*** | 2.04 to 2.99  2.38 to 3.49  2.91 to 4.23  4.75 to 6.31 |
| **Composite** | -0.11*** | -0.12 to -0.10 | 0.14*** | 0.13 to 0.15 | 0.04*** | 0.03 to 0.05 | 2.06*** | 1.87 to 2.24 |
| **Income (complete cases)**  Quartile 1 (£50,000+)  Quartile 2 (£25,000 to £49,999)  Quartile 3 (£13,500 to £24,999)  Quartile 4 (up to £13,499) | Ref  -0.20***  -0.32***  -0.45*** | -0.24 to -0.17  -0.36 to -0.29  -0.49 to -0.41 | Ref  <0.01  -0.08***0.02 | -0.03 to 0.04  -0.12 to -0.04  -0.02 to -0.06 | Ref  -0.17***  -0.30***  -0.26*** | -0.20 to -0.14  -0.33 to -0.26  -0.29 to -0.22 | Ref  -0.06  -0.45  0.57 | -0.81 to 0.70  -1.30 to 0.40  -0.27 to 1.41 |
| **Income (Missing data indicator)** |  |  |  |  |  |  |  |  |
| Quartile 1 (£50,000+) | Ref |  | Ref |  | Ref |  | Ref |  |
| Quartile 2 (£25,000 to £49,999) | -0.20*** | -0.24 to -0.17 | <0.01 | -0.04 to 0.04 | -0.17*** | -0.20 to -0.14 | -0.056 | -0.79 to 0.68 |
| Quartile 3 (£13,500 to £24,999) | -0.32*** | -0.36 to -0.28 | -0.08*** | -0.12 to -0.04 | -0.30*** | -0.33 to -0.26 | -0.451 | -1.28 to 0.38 |
| Quartile 4 (up to £13,499)  Missing | -0.45***  -0.34*** | -0.49 to -0.41  -0.37 to -0.31 | 0.02  -0.08*** | -0.03 to 0.06  -0.11 to -0.04 | -0.26***  -0.31*** | -0.29 to -0.22  -0.34 to -0.28 | 0.569  -1.394*** | -0.26 to 1.40  -2.08 to -0.71 |

**Note:** * significant at p<0.05; ** significant at p<0.01; and *** significant at p<0.001; Standardised coefficients are given for the composite score (mean 0.51 and SD 0.20)

**Supplementary Table B:** Model fit statistics (R-squared, AIC and BIC) and mean squared prediction error from 10-fold cross validation for the regression models presented in supplementary Table 1

|  | Unadjusted model | | |
| --- | --- | --- | --- |
|  | R^2^*100 | AIC/BIC | RMSE |
| Tenure ~ frequency | 2.577 | 182840.0/182866.8 | 1.237 |
| Employment ~ frequency | 0.749 | 183886.6/183913.4 | 1.238 |
| Income ~ frequency | 0.442 | 184062.9/184107.6 | 1.240 |
| Income^*^ ~ frequency | 1.561 | - - - - - - - - - - - - - - | 1.260 |
| Income^#^ ~ frequency | 1.180 | 185066.6/185120.1 | 1.272 |
| Education ~ frequency | 1.215 | 183625.0/183678.6 | 1.239 |
| Car ownership ~ frequency | 0.024 | 184296.9/184323.7 | 1.243 |
| Social grade ~ frequency | **3.062** | **182561.8/182615.4** | **1.232** |
| Composite ~ frequency | 0.763 | 183879.1/183905.9 | 1.238 |
| Tenure ~ quantity | **2.272** | **192400.4/192427.2** | **1.335** |
| Employment ~ quantity | 0.734 | 193279.4/193306.2 | 1.346 |
| Income ~ quantity | 0.068 | 193658.5/193703.2 | 1.350 |
| Income^*^ ~ quantity | 0.058 | - - - - - - - - - - - - - - | 1.377 |
| Income^#^ ~ quantity | 0.083 | 194601.5/194655.0 | 1.395 |
| Education ~ quantity | 2.122 | 192489.6/192543.3 | 1.336 |
| Car ownership ~ quantity | 0.089 | 193644.6/193671.4 | 1.350 |
| Social grade ~ quantity | 0.819 | 193234.4/193288.1 | 1.349 |
| Composite ~ quantity | 1.120 | 193060.4/193087.2 | 1.343 |
| Tenure ~ binge drinking | 0.988 | 169534.6/169561.4 | 1.090 |
| Employment ~ binge drinking | 1.100 | 169470.9/169497.7 | 1.089 |
| Income ~ binge drinking | 0.128 | 170023.1/170067.8 | 1.095 |
| Income^*^ ~ binge drinking | 0.959 | - - - - - - - - - - - - - - | 1.111 |
| Income^#^ ~ quantity | 0.980 | 168638.6/168692.2 | 0.979 |
| Education ~ binge drinking | **1.300** | **169359.6/169413.2** | **1.090** |
| Car ownership ~ binge drinking | 0.032 | 170075.5/170102.3 | 1.095 |
| Social grade ~ binge drinking | 0.139 | 170018.5/170072.1 | 1.095 |
| Composite ~ binge drinking | 0.159 | 170004.0/170030.8 | 1.095 |
| Tenure ~ weekly units | 1.258 | 510720.1/510746.9 | 22.528 |
| Employment ~ weekly units | 0.317 | 511254.7/511281.6 | 22.646 |
| Income ~ weekly units | 0.067 | 511397.5/511442.2 | 22.674 |
| Income^*^ ~ weekly units | 0.060 | - - - - - - - - - - - - - - | 27.837 |
| Income^#^ ~ quantity | 0.062 | 524722.9/524776.4 | 27.255 |
| Education ~ weekly units | **1.132** | **510685.0/510738.6** | **22.531** |
| Car ownership ~ weekly units | 0.078 | 511389.3/511416.1 | 22.673 |
| Social grade ~ weekly units | 0.484 | 511163.0/511216.7 | 22.666 |
| Composite ~ weekly units | 0.821 | 510969.2/510996.1 | 22.588 |

* Sensitivity analysis: complete cases only for income; ^#^ Sensitivity analysis: missing data indicator; AIC and BIC are not given for the complete case analysis as they both depend on sample size and can therefore not be compared to the other results where sample size is larger due to multiple imputation of missing values; adjusted R^2^ corrects for differences in sample size; RMSE is more valid for the main analysis due to the larger sample size[^49^](#_ENREF_49)

**Supplementary Table C:** Results of the ridge regression at optimal values of lambda (unadjusted for sex, age and ethnicity)

|  | Frequency of alcohol consumption | | Quantity of alcohol consumption | | Binge drinking frequency | | Weekly unit consumption | |
| --- | --- | --- | --- | --- | --- | --- | --- | --- |
|  | β | 95%CI | β | 95%CI | β | 95%CI | β | 95%CI |
| ***Tenure***  Owns home  Does not own home | Ref  -0.24* | -0.27 to -0.22 | Ref  0.24* | 0.21 to 0.26 | Ref  0.12* | - 1. to 0.14 | Ref  1.83* | 1.40 to 2.26 |
| ***Employment***  In full time work  Not in full time work | Ref  0.22* | 0.20 to 0.24 | Ref  -0.15* | -0.17 to -0.12 | Ref  -0.11* | -0.13 to -0.09 | Ref  -0.96* | -1.36 to -0.55 |
| ***Income***  Quartile 1 (£50,000+)  Quartile 2 (£25,000 to £49,999)  Quartile 3 (£13,500 to £24,999)  Quartile 4 (up to £13,499) | Ref  0.04*  -0.02  -0.10* | 0.02 to 0.07  -0.05 to 0.01  -0.13 to -0.06 | Ref  0.04*  -0.02  0.02 | - 1. to 0.07   -0.05 to 0.01  -0.02 to 0.05 | Ref  0.03*  -0.04*  -0.01 | <0.01 to 0.05  -0.07 to -0.01  -0.04 to 0.02 | Ref  0.34*  -0.15  0.33 | -0.11 to 0.78  -0.71 to 0.41  -0.27 to 0.92 |
| ***Education***  University  A level and equivalent  GCSE/vocational  Other/still studying  No post 16 qual | Ref  -0.11*  -0.11*  0.02*  -0.10* | -0.14 to -0.08  -0.14 to -0.09  0.06 to 0.02  -0.13 to -0.06 | Ref  0.24*  0.08*  -0.01  -0.08* | 0.21 to 0.27  0.05 to 0.11  -0.05 to 0.04  -0.12 to -0.04 | Red  0.11*  <0.01  -0.03  -0.12* | 0.09 to 0.14  -0.02 to 0.02  -0.06 to <0.01  -0.15 to -0.08 | Ref  1.98*  0.53*  -0.18  0.72* | 1.45 to 2.50  0.02 to 1.03  -0.89 to 0.54  -1.41 to -0.04 |
| ***Car ownership***  Owns car  Does not own car | Ref  -0.03 | Ref  -0.06 to <0.01 | Ref  0.06* | - 1. to 0.09 | Ref  0.03* | 0.01 to 0.05 | Ref  0.62* | - 1. to 1.13 |
| ***Social-grade***  AB  C1  C2  D  E | Ref  -0.10*  -0.16*  -0.28*  -0.30* | -0.12 to -0.07  -0.20 to -0.13  -0.32 to -0.24  -0.35 to -0.26 | Ref  0.02  0.06*  0.06*  0.19* | -0.01 to 0.05  0.03 to 0.09  0.02 to 0.10  0.14 to 0.24 | Ref  0.03*  <0.01  -0.02  0.01* | 0.01 to 0.05  -0.03 to 0.03  -0.05 to 0.01  0.03 to 0.05 | Ref  0.20  0.35  0.55  1.33* | -0.26 to 0.65  -0.22 to 0.92  -0.15 to 1.24  0.51 to 2.16 |

**Note:** standard errors for ridge regression are biased to allow accurate estimation of coefficients; * significant at p<0.05

**Supplementary File A:** Choosing the ridge regression model

The results of the unadjusted ridge regression for predicting frequency of alcohol consumption at different values of Lambda are given in supplementary Figure 1 (a). Supplementary Figure 1 (b), (c) and (d) also give the results of the ridge regression at different values of Lambda for quantity of alcohol consumption, frequency of weekly binge drinking and mean weekly unit consumption. Figures 2 (a), (b), (c) and (d) show the same results for the models adjusting for age, gender and ethnicity. Moving a short distance from the coefficients for the standard regression (x=0), leads to a rapid decrease in absolute values of the coefficients. Thus, it is clear from this that the standard regression coefficients were overestimating and were unstable.

The optimal Lambda, when the coefficients stabilised, for frequency was identified as 0.40 for the unadjusted and 0.21 for the adjusted model, for quantity it was identified as 0.87 for the unadjusted and 0.16 for the adjusted model, and for weekly binge drinking as 0.98 for the unadjusted and 0.14 for the adjusted model. The optimal Lambda for weekly number of units was identified as 43.47 for the unadjusted model and 7.02 for the adjusted model.


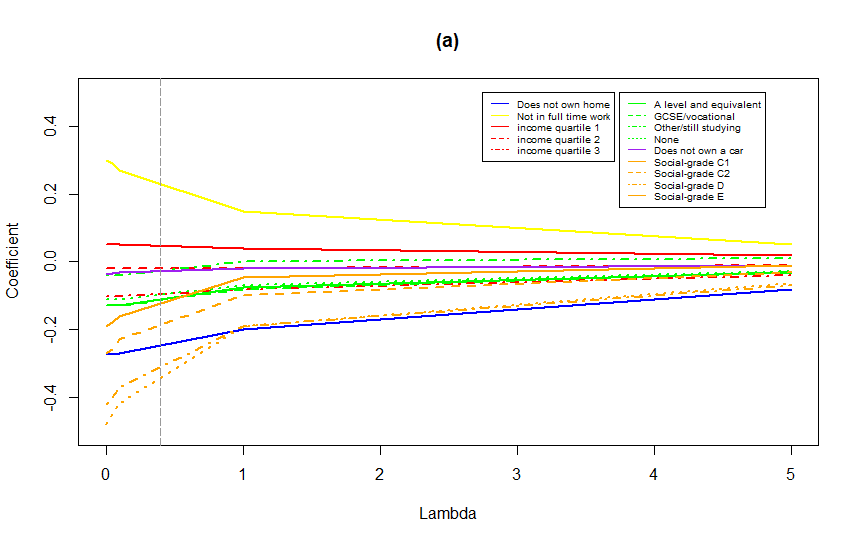


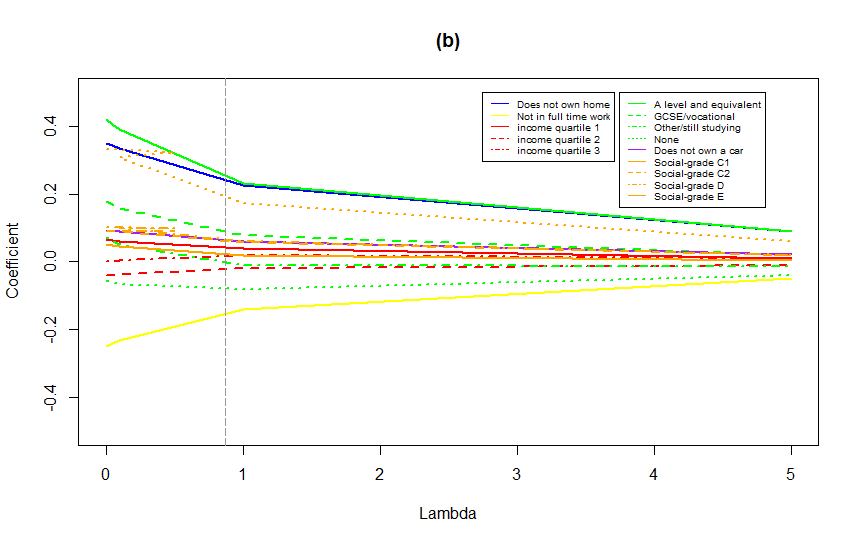


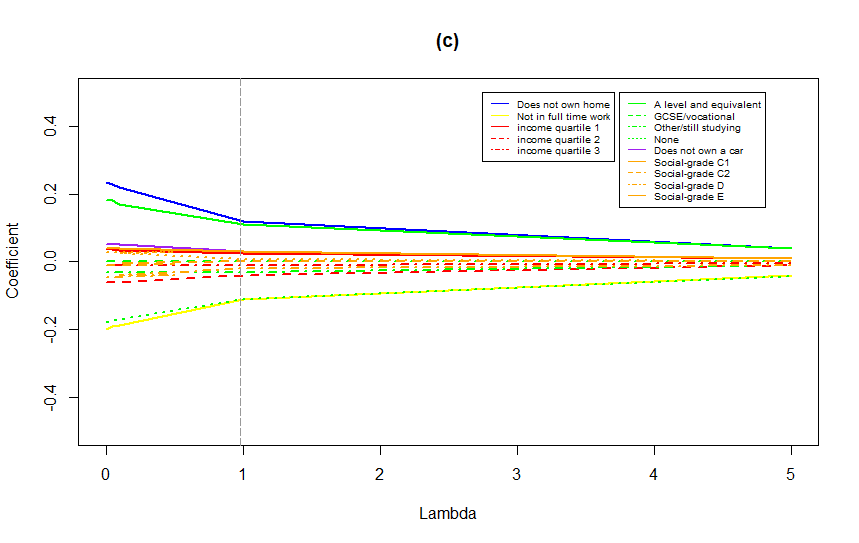


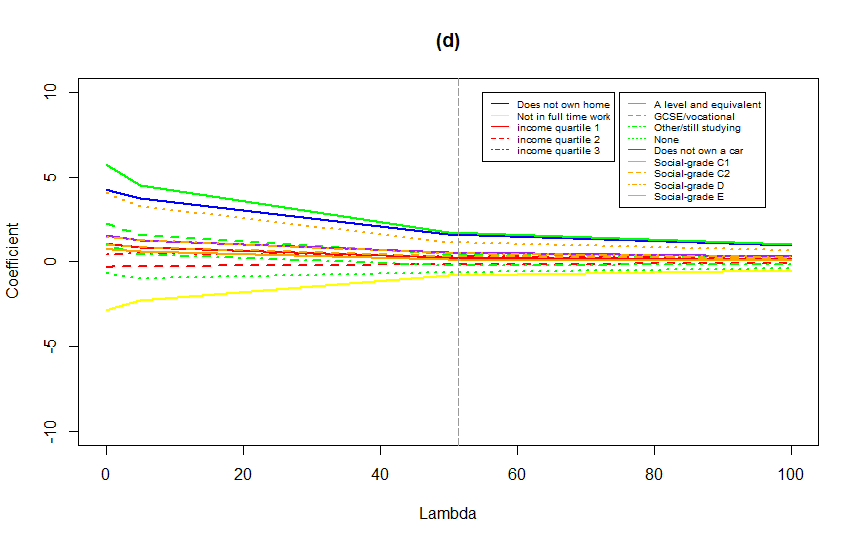


**Supplementary Figure A:** Results of the ridge regression at different values of Log(Lambda) for predicting a) frequency, b) quantity and c) frequency of binge drinking (unadjusted)

Note: grey dash line shows point of coefficient stabilisation


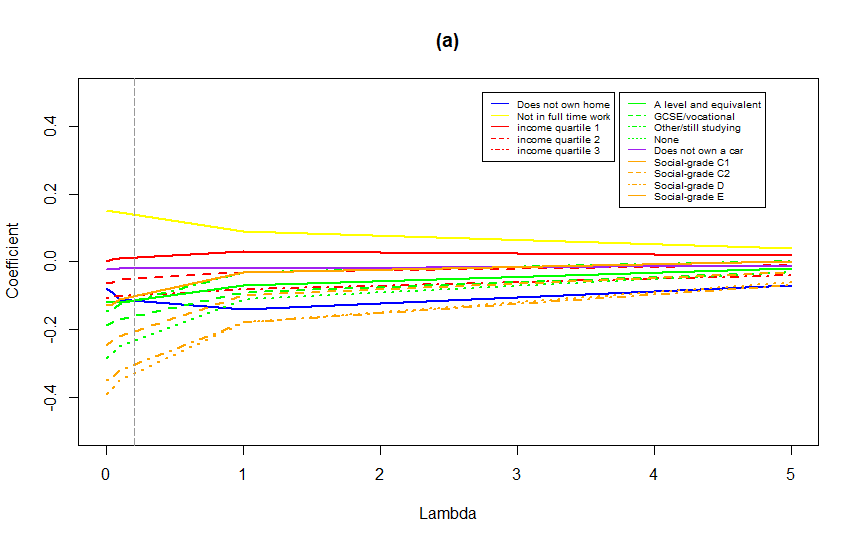


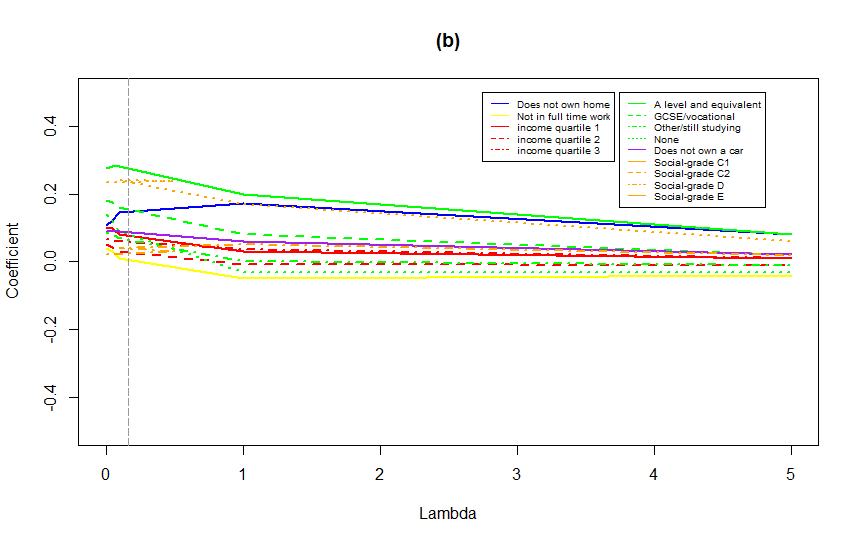


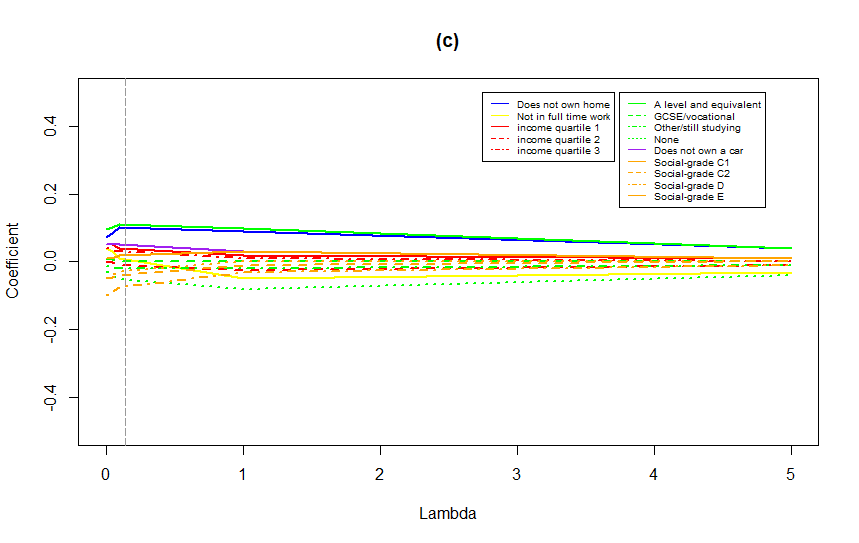


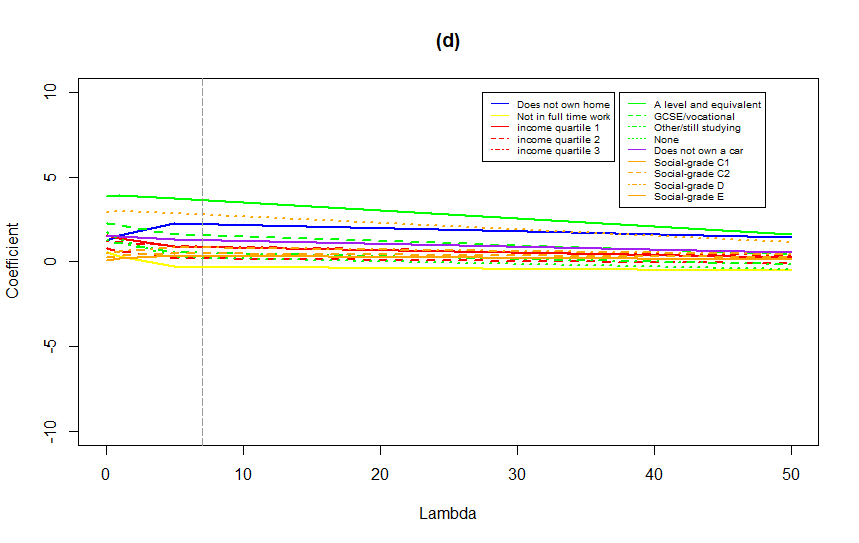


**Supplementary Figure B:** Results of the ridge regression at different values of Log(Lambda) for predicting a) frequency, b) quantity and c) frequency of binge drinking (adjusted)

Note: grey dash line shows point of coefficient stabilisation
